# Effects of ultrafine particles-induced oxidative stress on Clara cells in allergic lung inflammation

**DOI:** 10.1186/1743-8977-7-11

**Published:** 2010-04-26

**Authors:** Francesca Alessandrini, Ingrid Weichenmeier, Erik van Miert, Shinji Takenaka, Erwin Karg, Cornelia Blume, Martin Mempel, Holger Schulz, Alfred Bernard, Heidrun Behrendt

**Affiliations:** 1 Division of Environmental Dermatology and Allergy, Helmholtz Zentrum/Technische Universität München, Helmholtz Zentrum München, German Research Center for Environmental Health (GmbH), Ingolstaedter Landstrasse 1, 85764 Neuherberg, Germany, ZAUM Center for Allergy and Environment, Biedersteinerstrasse 29, 80802 Munich, Germany; 2 Focus Network Nanoparticles and Health (NanoHealth), Helmholtz Zentrum München, German Research Center for Environmental Health (GmbH), Ingolstaedter Landstrasse 1, 85764 Neuherberg, Germany; 3 Laboratory of Toxicology and Applied Pharmacology, Faculty of Medicine, Catholic University of Louvain, 53.02 Avenue E. Mounier, B-1200 Brussels, Belgium; 4 Institute of Lung Biology and Disease, Helmholtz Zentrum München, German Research Center for Environmental Health (GmbH), Ingolstaedter Landstrasse 1, 85764 Neuherberg, Germany; 5 Institute of Ecological Chemistry, Helmholtz Zentrum München, German Research Center for Environmental Health (GmbH), Ingolstaedter Landstrasse 1, 85764 Neuherberg, Germany

## Abstract

**Background:**

Clara cell protein (CC16), the main secretory product of bronchiolar Clara cells, plays an important protective role in the respiratory tract against oxidative stress and inflammation. The purpose of the study was to investigate the role of elemental carbon ultrafine particles (EC-UFP)-induced oxidative stress on Clara cells and CC16 in a mouse model of allergic lung inflammation.

**Methods:**

Ovalbumin (OVA)-sensitized mice were exposed to EC-UFP (507 μg/m^3 ^for 24 h) or filtered air immediately prior to allergen challenge and systemically treated with N-acetylcysteine (NAC) or vehicle prior and during EC-UFP inhalation. CC16 was measured up to one week after allergen challenge in bronchoalveolar lavage fluid (BALF) and in serum. The relative expression of CC16 and TNF-α mRNA were measured in lung homogenates. A morphometrical analysis of mucus hypersecretion and electron microscopy served to investigate goblet cell metaplasia and Clara cell morphological alterations.

**Results:**

In non sensitized mice EC-UFP inhalation caused alterations in CC16 concentration, both at protein and mRNA level, and induced Clara cell hyperplasia. In sensitized mice, inhalation of EC-UFP prior to OVA challenge caused most significant alterations of BALF and serum CC16 concentration, BALF total protein and TNF-α relative expression compared to relevant controls; their Clara cells displayed the strongest morphological alterations and strongest goblet cell metaplasia occurred in the small airways. NAC strongly reduced both functional and morphological alterations of Clara cells.

**Conclusion:**

Our findings demonstrate that oxidative stress plays an important role in EC-UFP-induced augmentation of functional and morphological alterations of Clara cells in allergic lung inflammation.

## Background

There is rapidly increasing epidemiological evidence associating respiratory health effects and exposures to ultrafine particles (UFP) in a susceptible population, such as elderly, young children, and people with pre-existing respiratory conditions [1]. For adult asthmatics, ambient levels of UFP (aerodynamic diameter <0.1 μm) were found to have a higher specific toxicological role compared to larger particles [2]. UFP, due to their large surface area, have enhanced capabilities to produce reactive oxygen species [3].

Clara cells are non-ciliated secretory epithelial cells lining the pulmonary airways, distinct from mucous and serous secretory cells in morphology and secretory products [4]. In rodents Clara cells represent the most frequent secretory cell population of proximal and distal airways [5]. Their function is mainly to protect the respiratory tract. Clara cells act as stem cells in the repair of bronchial and bronchiolar epithelium, have a high xenobiotic transformation capacity and secrete several proteins with important biological activities [6]. The main secretory molecule is a 16 kD protein (termed CC16, CC10, or CCSP), which is found in the electron dense, ovoid secretory granules, within the endoplasmic reticulum of Clara cells [7] and in the lining fluid throughout the conducting airways [8]. The immunomodulatory activity of CC16 has been well documented [9,10]. CC16 can inhibit phospholipase A2 activity [11], while inflammatory cytokines like TNF-α can modulate CC16 production [12]. In humans CC16 has been successfully used as a marker of alveolo-capillary barrier permeability [13-15]. Investigations of Clara cells response to inhaled particulates reflect the toxic effects of these particles to the respiratory tract [16]. Previous studies have reported alterations of CC16 concentrations in BALF and serum following exposure to cigarette smoke, ozone or DEP [17-19]. CC16 knockout mice showed aggravated early pro-inflammatory response to oxidant challenge [20,21], indicating a protective role of CC16 against acute oxidative stress.

Allergic asthma is characterized by airway hyperresponsiveness, airway inflammation, increased mucus production and lower antioxidant defenses [22]. The role of Clara cells and their secretory products in asthma is still controversial. Clara cells seem to exert protective effects against the development of the disease [23-26]; conversely, they are involved in allergen-induced mucus production [27,28]. We recently showed that particle-induced oxidative stress plays an important role in UFP-induced exacerbation of allergic airway inflammation [29].

The aim of the present study was to investigate the role of particle-induced oxidative stress on functional and morphological alterations of Clara cells in a mouse model of lung allergic inflammation. We measured serum and BALF CC16 concentration, CC16 relative expression in the lung in parallel to its activator TNF-α, and evaluated morphological alterations of Clara cells and goblet cell hyperplasia with and without the addition of the antioxidant NAC.

## Materials and methods

### Animals and materials

Five to seven-week-old Balb/c mice (Charles River, Sulzfeld, Germany) were housed in individually ventilated cages (VentiRack™, cage type CU-31, BioZone, Ramsgate, Kent, UK) and received a standard pellet diet and water *ad libitum*. The study was conducted under federal guidelines for the use and care of laboratory animals and was approved by the Government of the District of Upper Bavaria. All chemicals were purchased from Sigma-Aldrich Chemie, Deisenhofen, Germany, unless otherwise specified.

### Allergen sensitization/challenge protocol

Mice were sensitized by repetitive intraperitoneal injections of 1 μg OVA (grade VI; Sigma Aldrich Chemie)/Alum (2.5 mg; Pierce Chemical Co, Rockfort, IL, USA) in phosphate buffered saline (PBS) on days 0, 7, 14, 28, 42, 56. Blood samples were taken before and after sensitization. OVA-specific IgE and IgG1 were measured in plasma samples by ELISA as described previously [30]. OVA/alum sensitized mice (day 63), compared to non-sensitized controls, were characterized by high titers of OVA-specific IgE (24.2 ± 1.8 vs 0.1 ± 0.01 μg/ml) and OVA-specific IgG1 (1392.6 ± 182.6 vs <0.1 μg/ml). On day 70 the mice were aerosol-challenged for 20 min with 1%-LPS-depleted [30] OVA in PBS or with PBS alone delivered by Pari-Boy nebulizer (Pari GmbH, Starnberg, Germany).

### EC-UFP production, characterization and exposure

EC-UFP were generated by electric spark discharge (model GFG1000, Palas GmbH, Karlsruhe, Germany) using agglomerated carbon particles as previously described [29,30]. EC-UFP average size distributions were characterized by a count median diameter of 51.7 nm (geometric standard deviation = 1.54) and an average mass median diameter of 84.7 nm. Spark generated EC-UFP are considered to be structurally similar and form aggregates similarly to the primary particles of modern diesel emissions [31], but with extremely low condensed organic matter. Organic carbon content of EC-UFP was estimated less than 5% by evolved gas analysis and Fourier transform analysis [32]. Spark generated EC-UFP are then considered to be relatively inert particles compared to diesel exhaust or concentrated atmospheric particles. We chose them for the inhalation exposure because of their defined chemical composition and stable physical characteristics during the 24 h exposure experiment.

Details of animal exposures were described previously [29]. All EC-UFP exposures used for this study were performed for 24 h with a mass concentration of 507 μg/m^3^, which corresponds to an average particle number concentration of 9.3 × 10^6^/cm^3^.

### Experimental design

**Figure 1:**
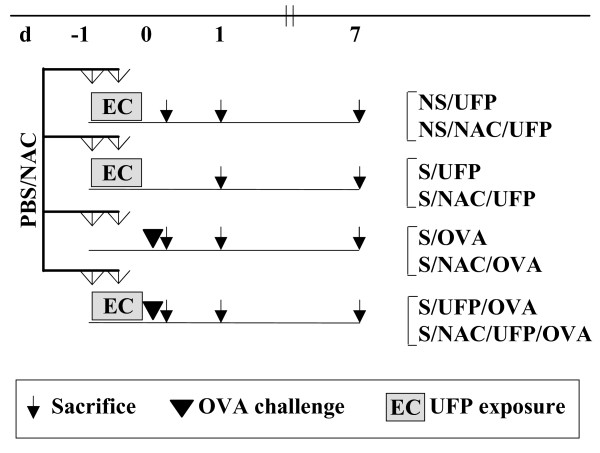
**Experimental groups (left) and designation (right)**. EC, 24 h exposure to elemental carbon UFP (507 μg/m^3^); open arrows, 2× intraperitoneal injection with N-acetylcysteine; black triangle, OVA aerosol challenge; ↓, sacrifice, BAL, histology and electron microscopy. S, sensitized; NS, non sensitized; OVA, challenged with aerosolized OVA; UFP, exposed to EC-UFP.

A schematic representation of the study protocol is shown in Fig. 1.

In order to evaluate the effect of EC-UFP-induced oxidative stress on functional and morphological alterations of Clara cells, sensitized (S) and subsequently challenged mice (OVA) were exposed to EC-UFP with (S/NAC/UFP/OVA) and without NAC treatment (S/UFP/OVA). NAC was administered with two intraperitoneal injections (250 mg/kg body weight in PBS, 200 μl), 1× shortly before and 1× close to mid-EC-UFP exposure. OVA sensitized and subsequently challenged mice exposed to filtered air (S/OVA) and non sensitized (NS) or sensitized mice exposed to EC-UFP (NS/UFP, S/UFP) with and without NAC treatment served as controls. In addition, untreated animals served as baseline controls (n = 8). Before bronchoalveolar lavage (BAL), blood samples were taken from retro orbital plexus and centrifuged. Serum aliquots were stored at -80°C. BAL was performed at 0, 1, or 7 days after OVA challenge, or after termination of EC-UFP exposure (n = 4/time point). After centrifugation, BALF was stored at -80°C for subsequent analysis. Lungs were stored in formalin for histology/snap frozen in liquid nitrogen and stored at -80°C for isolation of RNA. Airway hyperresponsiveness was evaluated by body plethysmography in untreated animals, or 24 h after OVA challenge in S/OVA, S/UFP/OVA and S/NAC/UFP/OVA (n = 5-7).

### Bronchoalveolar lavage (BAL) analysis

Airways were lavaged five times with 0.8 ml PBS. Aliquots of cell-free BAL fluid were assayed in duplicate for total protein (Coomassie Protein Assay, Pierce Chemical Co.) and for IL-5, IFN-γ and IL-13 by two site ELISAs using antibodies from BD Biosciences (IL-5 and IFN-γ, Heidelberg, Germany) and BioSource (IL-13, Camarillo, CA, USA) as suggested by the manufacturer. Viability and yield of BAL cells was quantified via trypan blue exclusion in a hemocytometer. Differential BAL cell count (400 cells/sample) was performed on cytospins (600 rpm for 10 min) fixed and stained with Diff-Quick (Dade Behring, Marburg, Germany).

### Determination of CC16 in BALF and serum

Cell-free BALF and serum aliquots were assayed in duplicate for CC16 by an automated latex immunoassay using a polyclonal rabbit antibody raised against rat CC16 and applicable to both rat and mouse CC16 analysis [33].

### RNA extraction and real time RT-PCR

Real-time RT-PCR analysis was performed in the lungs retrieved 1 day after EC-UFP exposure/allergen challenge. After homogenization, total RNA was isolated from total lung tissue using the RNeasy Mini kit (Qiagen GmbH, Hilden, Germany) as per supplier's instructions. First strand cDNA synthesis was performed using the iScript cDNA synthesis kit (BioRad Laboratories GmbH, München, Germany). Quantitative PCR was performed using TaqMan^® ^Gene Expression Assays (CC16: Mm01230908_m1; TNF-α: Mm00443258_m1; 18S: Hs99999901_s1) and TaqMan^® ^Universal PCR Master Mix in an ABIprism7000 Sequence Detection System (Applied Biosystems, Darmstadt, Germany). The mRNA expression levels were normalised to the according expression levels of the housekeeping gene 18S and the mean expression levels of the untreated group using the ΔΔC_T _method.

### Histological evaluation and morphometrical analysis

After BAL the lungs were removed and fixed in 10% buffered formalin. The left lobe was used for histopathology. After paraffin embedding, 3.5 μm sections were stained with hematoxylin-eosin (H&E) and periodic acid Schiff (PAS). In order to evaluate goblet cell metaplasia and mucus hypersecretion, we performed a morphometrical analysis of volume density of PAS positive material (double square lattice test system E80 [34] and Optimas software package, Washington, USA) in cross sections of small (circumference ≤ 800 μm), and large (circumference >800 μm) airways of the following groups: NS/UFP, S/OVA, S/UFP/OVA and S/NAC/UFP/OVA. The data are expressed as percentage of PAS positive material/total epithelial cells.

### Transmission (TEM) and scanning (SEM) electron microscopy

For electron microscopy, lungs were fixed by intratracheal perfusion with 2.5% glutaraldehyde in 0.1 M cacodylate buffer (pH 7.4), (n = 2/time point/group). For TEM peripheral and central sections of the right lung were minced, for SEM the left lung was cut longitudinally; all lung samples were fixed for further 4-7 days in glutaraldehyde and postfixed in 1% osmiumtetroxide in cacodylate buffer for 1 h at 4°C. For TEM the samples were embedded in EMbed 812 (Science Services, München, Germany). Semithin sections (500 nm) stained with Richardson solution (methylenblue, azur II) were used for selecting the portion suitable for electron microscopy. Then, ultrathin sections (silver coloured) were cut, mounted on formvar-coated 75-mesh nickel grids, double-stained with uranyl acetate and lead citrate and observed in a transmission electron microscope (JEOL 1210, Tokyo, Japan) at 80 kV. Photographs were taken using Kodak 4489 films.

For SEM, the specimens were rinsed twice in cacodylate buffer, dehydrated in an ascending series of ethanols and critical point dried. The lungs were then mounted on plates using Leit-Tabs (Plano, Wetzlar, Germany), sputter-coated with 10 nm gold and examined in a scanning electron microscope (JEOL 6300, Tokyo, Japan) working at 15 kV. Photographs were taken using a digitalized image processing system.

### Lung function tests

Lung function tests were performed 24 h after allergen challenge in unrestrained animals using whole body plethysmography (Buxco^® ^Electronics, Sharon, Connecticut, USA). For whole body plethysmography, the 'enhanced pause' (PenH) determined before and after methacholine exposure was applied as an index of airway hyperresponsiveness (AHR), as previously described [29,30]. The doses of methacholine aerosol (M1-M5) delivered to the mice were the following: 10 mg/ml methacholine nebulized for 1, 2 and 4 minutes (M1-M3), followed by 40 mg/ml methacholine for 2 and 4 minutes (M4-M5). A data recording interval of 3 min was introduced after each methacholine level. The mean of the PenH values determined in the 2^nd ^and 3^rd ^min was used for quantifying AHR.

### Statistical analysis

Data were expressed as mean ± SD. For statistical evaluation, one-way analysis of variance (ANOVA) with post-hoc Scheffé Test (applied for BAL cells and BALF total protein, cytokines and morphometrical analysis) and Least Significant Difference-Test (LSD) (applied for CC16 analysis in BALF and serum, CC16 and TNF-α relative mRNA expression and airway hyperresponsiveness) comparisons were used (Statistica Stat Soft, 6.0). A p-value < 0.05 was considered to be significant.

## Results

### NAC reduced EC-UFP-induced alterations in CC16 concentrations in BALF and serum

**Figure 2:**
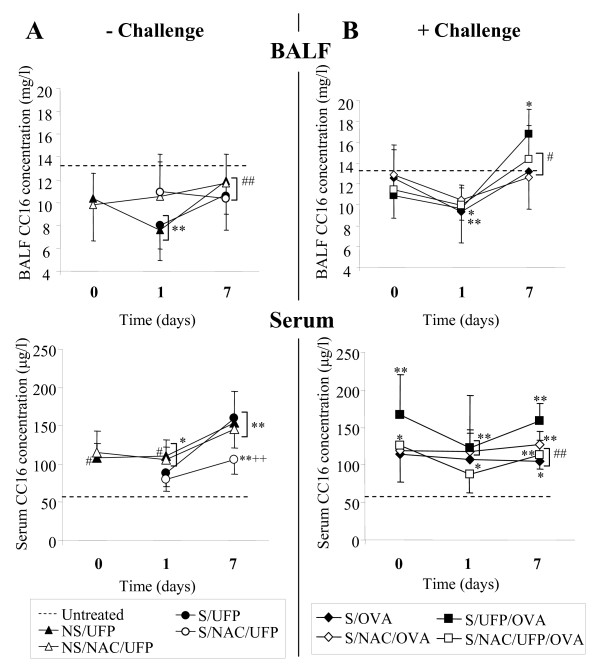
**BALF and serum CC16 concentration**. (**A**) Non sensitized (NS/UFP) and sensitized (S/UFP) mice exposed to EC-UFP, and treated with NAC (NS/NAC/UFP and S/NAC/UFP, respectively); (**B**) Sensitized and challenged mice (S/OVA), treated with NAC (S/NAC/OVA), or exposed to EC-UFP 24 h prior to OVA challenge (S/UFP/OVA), and treated with NAC (S/NAC/UFP/OVA). NAC treatment was performed prior to and close to mid EC-UFP exposure. Untreated mice served as baseline controls (dotted line). Data presented as mean ± SD (n = 4-7/time point). *p < 0.05, **p < 0.01 vs untreated; ^++^p < 0.01 vs S/UFP;^#^p < 0.05, ^##^p < 0.01 NS/UFP, S/UFP, S/OVA, S/NAC/UFP/OVA vs S/UFP/OVA.

The concentrations of CC16 in BALF and serum of untreated mice are shown in Fig. 2 by a dotted line. Non sensitized and sensitized mice exposed to EC-UFP for 24 h (NS/UFP, S/UFP) showed a significant decrease in CC16 in BALF at 1-day time point (Fig. 2a, upper panel). EC-UFP-induced decrease in BALF CC16 concentrations were abrogated by NAC treatment (NS/NAC/UFP; S/NAC/UFP). One week after EC-UFP inhalation, BALF CC16 concentrations almost reached the control level. In sensitized mice, OVA challenge alone caused a transitional reduction in CC16 content in BALF at 1-day time point (S/OVA, Fig. 2b, upper panel). NAC minimally reduced OVA-induced CC16 alterations (S/NAC/OVA). Sensitized mice exposed to EC-UFP inhalation prior to allergen challenge showed an initial significant reduction in BALF CC16, followed by a significant increase in CC16 BALF content at the 7-day time point compared to untreated animals, NS/UFP, S/UFP and to S/OVA (S/UFP/OVA, Fig. 2b, upper panel). NAC treatment significantly reduced this increase (S/NAC/UFP/OVA).

In contrast to BALF CC16 concentrations, serum CC16 concentrations increased significantly following exposure to EC-UFP in both non sensitized and sensitized mice (Fig. 2a, bottom panel). Increased CC16 concentrations in serum were detectable also one week after EC-UFP inhalation. NAC reduced serum CC16 concentrations in sensitized mice exposed to EC-UFP at the 7 days time point (Fig. 2a, bottom panel). In S/OVA serum CC16 concentrations were increased compared to untreated animals at all time points of evaluation (S/OVA, Fig. 2b, bottom panel) and NAC treatment had no effect on serum CC16 concentration (S/NAC/OVA). In sensitized animals, EC-UFP inhalation followed by allergen challenge further increased serum CC16 concentration (S/UFP/OVA, Fig. 2b, bottom panel). NAC treatment significantly reduced the EC-UFP-induced increased serum CC16 concentrations (S/NAC/UFP/OVA).

### NAC reduced EC-UFP-induced alterations in BALF total protein concentration, cytokines and airway inflammation

**Figure 3:**
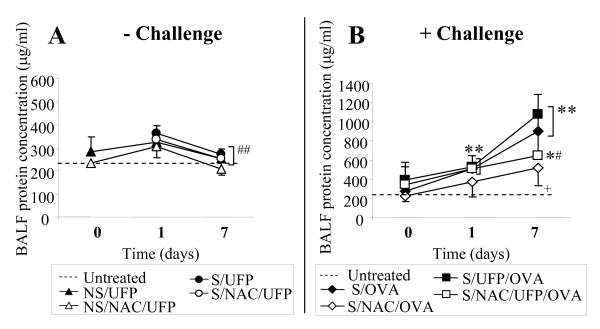
**BALF total protein concentration**. (**A**) Non sensitized (NS/UFP) and sensitized (S/UFP) mice exposed to EC-UFP, and treated with NAC (NS/NAC/UFP and S/NAC/UFP, respectively); (**B**) Sensitized and challenged mice (S/OVA), treated with NAC (S/NAC/OVA), or exposed to EC-UFP 24 h prior to OVA challenge (S/UFP/OVA), and treated with NAC (S/NAC/UFP/OVA). NAC treatment was performed prior to and close to mid EC-UFP exposure. Untreated mice served as baseline controls (dotted line). Data presented as mean ± SD (n = 4-8/time point). *p < 0.05, **p < 0.01 vs untreated; ^+^p < 0.05 vs S/OVA; ^#^p < 0.05, ^##^p < 0.01 NS/UFP, S/UFP, S/OVA, S/NAC/UFP/OVA vs S/UFP/OVA.

The concentration of BALF total protein in untreated mice is shown in Fig. 3 by a dotted line. In sensitized mice, OVA challenge alone caused an increase in BALF total protein concentration compared to control, which was significantly reduced by NAC treatment (Fig. 3b). A further increase of BALF total protein was detected in sensitized mice exposed to EC-UFP inhalation prior to OVA challenge (S/UFP/OVA) at the 7 days time point (Fig. 3b). NAC significantly reduced BALF total protein concentrations (S/NAC/UFP/OVA) at the 7 days time point. No induction of protein levels were observed in any of the non-challenged groups (NS/UFP, S/UFP, with and without NAC treatment, Fig. 3a).

In sensitized mice, EC-UFP inhalation prior to OVA challenge (S/UFP/OVA) caused a significant increase of BAL inflammatory infiltrate and BALF cytokines IL-5 and IL-13 7 days after OVA challenge, compared with sensitized and challenged mice exposed to filtered air (S/OVA); NAC treatment significantly reduced EC-UFP-induced augmentation of lung allergic inflammation (S/NAC/UFP/OVA), (See additional file 1: functional characterization of the mouse model). NAC non-significantly reduced airway inflammation in S/OVA mice (S/NAC/OVA, see additional file 1: functional characterization of the mouse model). No significant alterations in the IFN-γ concentration were observed in any of the groups (data not shown). Non sensitized mice exposed to EC-UFP did not develop an inflammatory infiltrate and showed no cytokine release (NS/UFP, see additional file 1: functional characterization of the mouse model).

### At the mRNA level, NAC failed to reduce EC-UFP-induced alterations of CC16, but reduced EC-UFP-induced TNF-α relative expression

**Figure 4:**
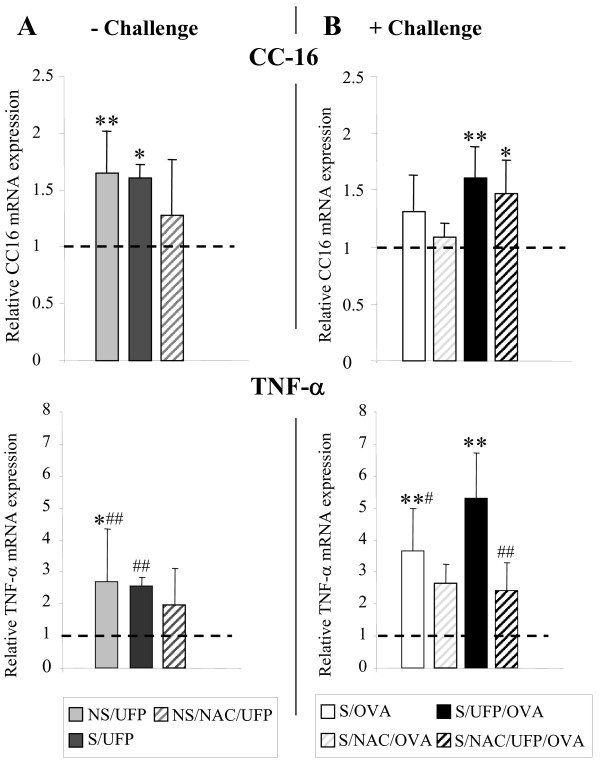
**CC16 and TNF-α relative mRNA expression in lung homogenates**. (**A**) Non sensitized (NS/UFP) and sensitized (S/UFP) mice exposed to EC-UFP, and NS/UFP treated with NAC (NS/NAC/UFP); (**B**) sensitized and challenged mice (S/OVA), treated with NAC (S/NAC/OVA), or exposed to EC-UFP 24 h prior to OVA challenge (S/UFP/OVA), and treated with NAC (S/NAC/UFP/OVA). NAC treatment was performed prior to and close to mid EC-UFP exposure. Untreated mice served as baseline controls (dotted line). The lungs were retrieved 24 h after EC-UFP/filtered air exposure. Data presented as mean ± SD (n = 3-6/group). *p < 0.05, **p < 0.01 vs untreated; ^#^p < 0.05, ^##^p < 0.01 NS/UFP, S/UFP, S/OVA, S/NAC/UFP/OVA vs S/UFP/OVA.

CC16 and TNF-α relative mRNA expression was evaluated at 1-day time point in lung homogenates. The relative mRNA expression of CC16 and TNF-α in untreated mice is shown in Fig. 4 by a dotted line. In sensitized and non sensitized mice EC-UFP exposure alone induced an increase in CC16 and TNF-α relative mRNA expression compared to untreated mice (Fig. 4a, upper and bottom panel respectively). In sensitized animals allergen challenge alone had no significant effects on relative CC16 mRNA expression, but significantly increased TNF-α expression (S/OVA, Fig. 4b, upper and bottom panel, respectively). In sensitized animals, EC-UFP exposure prior to allergen challenge increased CC16 mRNA expression similarly to NS/UFP and S/UFP (S/UFP/OVA, Fig. 4b, upper panel), whereas it significantly increased TNF-α expression compared to all other groups (S/UFP/OVA, Fig. 4b, bottom panel). NAC moderately reduced CC16 and TNF-α mRNA expression in non challenged animals (NS/NAC/UFP, Fig. 4a, striped bars). In sensitized and challenged animals NAC failed to reduce EC-UFP-induced increase in CC16 expression, but strongly reduced EC-UFP-induced increase in TNF-α mRNA expression (S/NAC/UFP/OVA, Fig. 4b, upper and bottom panel, respectively, striped bars).

### NAC strongly reduced EC-UFP-induced mucus hypersecretion in allergic animals

**Figure 5:**
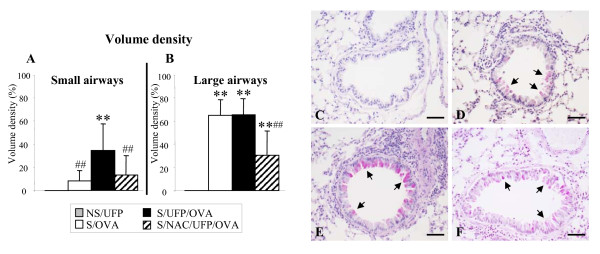
**Morphometrical analysis of volume density of PAS positive material**. NS/UFP, S/OVA, S/UFP/OVA and S/NAC/UFP/OVA lungs were retrieved 7 days after OVA challenge/EC-UFP inhalation and stained with PAS. (**A**) Small airways (circumference ≤ 800 μm); (**B**) Large airways (circumference >800 μm), n = 6-12/group. **p < 0.01 vs NS/UFP; ^##^p < 0.01 vs S/UFP/OVA. Representative samples of lungs stained with PAS showing small airways in: (**C**) Non sensitized mice exposed EC-UFP (NS/UFP); (**D**) Sensitized and challenged mice exposed to filtered air (S/OVA); (**E**) Sensitized mice exposed to EC-UFP 24 h prior to OVA challenge (S/UFP/OVA); (**F**) Sensitized mice exposed to EC-UFP 24 h prior to OVA challenge and treated with NAC (S/NAC/UFP/OVA). Arrows, mucus hypersecretion; scale bar, 50 μm.

In order to evaluate the degree of goblet cell metaplasia and mucus hypersecretion we performed a morphometrical analysis of PAS positive material in histological specimen retrieved 7 days after allergen challenge/EC-UFP inhalation. NS/UFP showed no PAS positive material (Fig. 5a, b, c). In sensitized animals allergen challenge alone had a significant effect on goblet cell metaplasia and mucus hypersecretion (Fig. 5a, b, d). EC-UFP exposure prior to allergen challenge increased goblet cell metaplasia and mucus hypersecretion in the small airways, but not in the large airways (Fig. 5a, b, e). NAC treatment prior and during EC-UFP inhalation significantly decreased goblet cell metaplasia and mucus hypersecretion both in small and large bronchi (Fig. 5a, b, f).

### NAC strongly reduced EC-UFP-induced morphological alterations in Clara Cells

**Figure 6:**
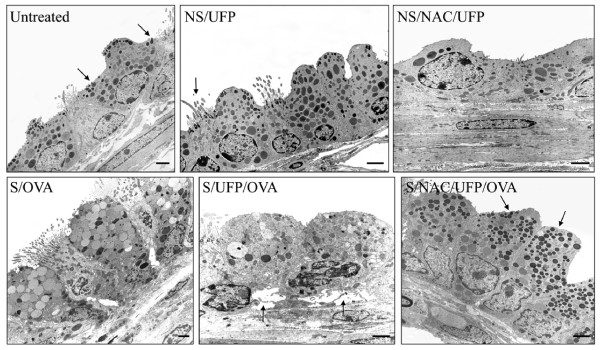
**Transmission electron microscopy**. Ciliated and non-ciliated (Clara) cells in the peripheral lung of normal control mice (**Untreated**). Merocrine secretions of electron-dense secretory granules (arrows) are recognized in the Clara cell exhibiting many mitochondria and developed smooth endoplasmic reticulum. Hyperplastic Clara cells in the lungs of non sensitized mice exposed to EC-UFP, (**NS/UFP**). The arrow points at a ciliated cell. NS/UFP treated with NAC (**NS/NAC/UFP**). OVA sensitized and challenged mice showing goblet cell metaplasia (**S/OVA**). Strong ultrastructural damage and detachment of metaplastic cells (arrows) is shown in the peripheral lungs of sensitized mice exposed to EC-UFP 24 h prior to OVA challenge (**S/UFP/OVA**). Clara cells of sensitized mice exposed to EC-UFP 24 h prior to OVA challenge and treated with NAC show preserved ultrastructure with secretory granules (arrows), (**S/NAC/UFP/OVA**). NAC treatment was performed prior to and close to mid EC-UFP exposure. Scale bar, 2 μm.

**Figure 7:**
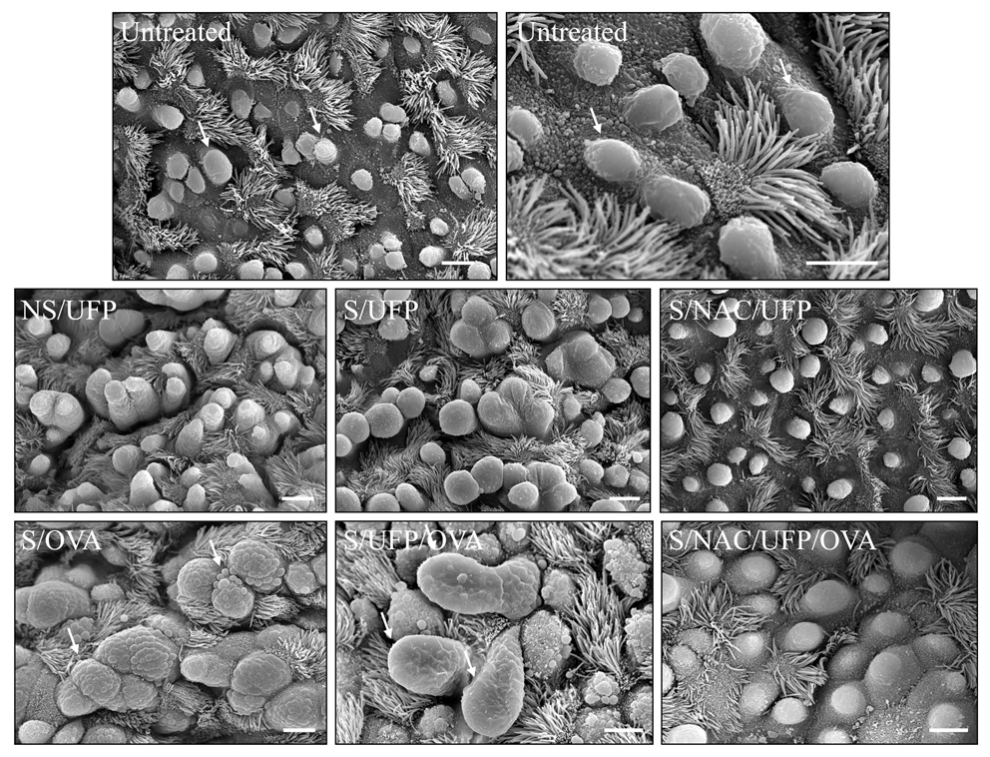
**Scanning electron microscopy**. Ciliated and non-ciliated (Clara) cells (arrows) in the bronchiolar epithelium of normal control mice (**Untreated**). Hyperplastic Clara cells in the lungs of EC-UFP-exposed non sensitized (**NS/UFP**) and sensitized mice (**S/UFP**). NAC treatment preserved Clara cell morphology (**S/NAC/UFP**). OVA sensitized and challenged mice showing goblet cell metaplasia (arrows, **S/OVA**). Highly hyperplastic Clara cells (arrows) between mucous cells in the bronchiolar epithelium of sensitized mice exposed to EC-UFP 24 h prior to OVA challenge (**S/UFP/OVA**). NAC treatment preserved Clara cell morphology (**S/NAC/UFP/OVA**). NAC treatment was performed prior to and close to mid EC-UFP exposure. Scale bar, 5 μm.

In the analysis of the samples performed by SEM and TEM, we focussed on the small airways (circumference ≤ 800 μm), one week time point. In untreated mice, Clara cells appeared as cuboidal cells with a central narrow projection into the airway lumen covered with short microvilli (Fig. 6, untreated). The upper third of the cell contained well organized arrays of smooth endoplasmatic reticulum. Two types of mitochondria could be observed; one small and elongated with cristae and the other large and usually round, with few or no cristae. Dense homogenous secretory granules (Fig. 6, untreated, arrows) bound by a single limiting membrane were located near the apex of the Clara cells most regularly immediate below the luminal membrane. Both merocrine and apocrine secretions were observed. Throughout the depth of the epithelium there were close connections between adjacent cells. No goblet cells could be detected. At the SEM, Clara cells from untreated mice appeared often arranged in a pattern forming lines. They showed dome-shaped apical projections into the lumen with rough surfaces (Fig. 7, untreated).

Following EC-UFP exposure, no particles could be detected within Clara cells. EC-UFP inhalation alone induced alterations in the morphology of Clara cells. In TEM as well as in SEM the Clara cells of the bronchioli exhibited hyperplasia and proliferation, so that the ciliated cells seemed to be reduced in number and appeared to be constricted (Fig. 6, NS/UFP, arrow; Fig. 7, NS/UFP). Sensitized animals exposed to EC-UFP showed the formation of clusters of 3 or 4 Clara cells close together (Fig 7, S/UFP). NAC reduced to a great extent these morphological alterations: no clusters were found, and Clara cell surface displayed the typical dome-shaped apical projections as in untreated mice (Fig. 6 and 7, NS/NAC/UFP and S/NAC/UFP).

In S/OVA mice, the bronchioli featured goblet cell metaplasia: the dense homogenous secretory granules of the Clara cells converted to mucus containing vesicles, some of them containing dark residues of the secret (Fig. 6, S/OVA). Signs of partial necrosis were also present. Primarily merocrine secretion of mucus was found. The vesicles in close proximity to the cell surface were bulging the plasma membrane. Due to this effect, at the SEM analysis the surface area of the transformed cells exhibited a vesicular pattern (Fig. 7, S/OVA).

NAC treatment reduced the ultrastructural modifications of the Clara cells of S/OVA mice (data not shown).

In sensitized animals, EC-UFP exposure prior to OVA challenge produced most profound ultrastructural damage and necrosis. Goblet cell metaplasia was more pronounced compared to S/OVA animals. In some areas no rudiments of the secretory granules could be detected and the abundant smooth endoplasmatic reticulum as well as the dimorphic mitochondria were entirely displaced by mucous vesicles. Widening of the intercellular spaces and detachment of the epithelium were frequently observed (Fig. 6, S/UFP/OVA, arrows). At the SEM analysis, a few highly hyperplastic Clara Cells were noted within goblet cells (Fig. 7, S/UFP/OVA, arrows).

NAC treatment extensively reduced the described morphological alterations. Over broad ranges of bronchioli Clara cells displayed normal features. Necrosis appeared to a striking less degree. Also at the SEM analysis, the surface of the Clara cells exhibited obvious less vesicular pattern (Fig. 6 and 7, S/NAC/UFP/OVA).

### NAC reduced EC-UFP-induced airway hyperresponsiveness

Airway hyperresponsiveness as measured by body plethysmography 24 h after OVA challenge showed significantly increased enhanced pause (PenH) following increasing methacholine concentrations in OVA sensitized animals exposed to EC-UFP prior to OVA challenge (S/UFP/OVA) compared to untreated; a close to significant increase in PenH was shown in comparison with sensitized and challenged mice exposed to filtered air (S/OVA). NAC reduced EC-UFP-dependent increase in airway hyperresponsiveness (See additional file 1: functional characterization of the mouse model).

## Discussion

Epidemiological and experimental studies suggest that anthropogenic air pollutants, in particular fine and ultrafine particulate matter, are important cofactors in the development of pulmonary health disorders, including pulmonary allergic disorders [30,35-37]. Clara cells are nonciliated secretory epithelial cells lining the pulmonary airways, whose main function is to protect the respiratory system from excessive inflammatory reactions [8]. In this study we investigated functional and morphological alterations of Clara cells in a mouse model of UFP-induced exacerbation of allergic lung inflammation. Our results showed that sensitized and challenged mice exposed to EC-UFP prior to allergen challenge showed a transient decrease, followed by a late increase of CC16 in BALF accompanied by a strong increase of CC16 in serum. BALF total protein concentration was also most increased in this group, confirming that the exudative response caused by the increased airway permeability plays an important role. Similarly to our results, exposure to cigarette smoke provoked a lower CC16 recovery in BALF and an increase in CC16 in serum [17]. In this study, the augmented albumin and total protein recovery in the BALF confirmed the increased particle-induced permeability of the lung/blood barrier. Interestingly, a recent study showed that cigarette smoke decreases transepithelial resistance resulting in a reduction of the epithelial barrier function [38]. We show similar effects using allergic mice, where lung injury caused by particle exposure and by allergic inflammation had synergistic effects. On the contrary, EC-UFP inhalation in non challenged non sensitized or sensitized mice had a transitional, though significant effect on CC16 concentrations in BALF, a significant effect on serum CC16 concentrations, but no effect on total protein recovery in BALF. Difference in particle characteristics, mainly the low toxicity of the relatively inert EC-UFP compared to cigarette smoke, characterized by a highly reactive gas phase and organics, might account for these discrepancies.

At the mRNA level, we show that EC-UFP alone stimulate CC16 expression in non sensitized and sensitized mice, independently from allergen challenge. This means that EC-UFP inhalation alone leads to increased intracellular storage of CC16 protein. While previous studies have reported that the mRNA level of CC16 in the respiratory tract is reduced after exposure to cigarette smoke, ozone, diesel exhaust and silicon carbide whiskers mineral fibers [17-19,39], the pulmonary response to intratracheal instillation of fine particles showed to increase CC16 mRNA expression [40]. This latter study is in line with our results. Altered expression of CC16 in the lung may depend on its key function. Most importantly, its anti-inflammatory activities include down-regulation of interferon-γ and tumor necrosis factor-alpha synthesis and/or biological activity [9], inhibition of IL-1β and of phospholipase A2 in the synthesis of arachidonic acid metabolites [11]. CC16 may therefore contribute to the regulation of an inflammatory response in the lung [41]. In this study we measured also the expression of TNF-α, an early-response multifunctional cytokine, produced mainly by monocytes and macrophages [42], which was shown to be synthesized in the lung following ultrafine particle exposure [43,44]. In addition to its pro-inflammatory characteristics, TNF-α was shown to stimulate human CC16 production [12]. In our model, EC-UFP inhalation alone increased TNF-α expression. Moreover, allergic inflammation and EC-UFP inhalation had synergistic effects on lung TNF-α expression.

Allergic asthma is a disease characterized by lower antioxidant defenses [22]. The role of Clara cells in asthma is still controversial. Studies in humans revealed decreased CC16 levels in BALF and serum of asthmatic patients compared to healthy individuals [23,25,45], suggesting that Clara cells may protect the lung against the development of the disease. Interestingly, the human gene for CC16 is localized within a chromosomal locus associated with regulation of inflammation and allergy [41] and an association has been found between a polymorphism in the CC16 gene and an increased risk of developing asthma [24]. In addition, experimental studies showed a regulation of the Th2 response by CC16 [26]. On the other hand, there is increasing evidence that Clara cells are essential in allergen-induced mucus production in the airway epithelium and that these cells undergo metaplasia to goblet cells, being responsible for the formation of the mucus plugs which characterize the asthmatic phenotype [27,28]. Thus, our mouse model represents a susceptible population to the EC-UFP-induced oxidative stress, where Clara cells seem to play an important role.

Oxidative stress is said to occur in a tissue or an organ when the normal balance between oxidants and antioxidants shifts in favor of oxidants, from either an excess of oxidants and/or depletion of antioxidants. Recently we have shown increased marker of oxidative stress isoprostane following EC-UFP inhalation alone; sensitized mice exposed to EC-UFP before OVA challenge showed highest lung isoprostane concentrations, NF-κB activation and airway hyperresponsiveness [29]. The role of CC16 in the protection against acute oxidative stress is well known. In fact, exposure to ozone was shown to increase serum CC16 concentrations [18] and CC16 -/- gene targeted mice showed increased susceptibility to hyperoxic injury and exaggerated inflammatory response compared to wild types [20,21]. In order to assess the role of oxidative stress in EC-UFP-induced alteration of Clara cells and CC16 concentration, we used NAC, a well-known thiol compound which acts directly as a free radical scavenger [46] and as a precursor of reduced glutathione (GSH) [47]. We chose NAC because it is relative innocuous, it has been widely used both *in vitro *and *in vivo *and it possess a short half life, therefore the antioxidant capabilities of NAC were limited only to the duration of EC-UFP exposure [48]. Our results show that EC-UFP-induced alterations of CC16 concentrations were significantly reduced by NAC treatment, indicating the role of oxidative stress in the induction of CC16 release. Similarly, the antioxidant strategy reduced TNF-α mRNA expression in whole lung homogenate. On the contrary, NAC did not reduce significantly CC16 mRNA expression, implying that the regulation of CC16 by NAC may function at the post-transcriptional level. The effect of NAC on CC16 concentrations in serum was slightly more inconsistent than in BALF. This could be explained by the large gradient across the air/blood barrier, where a small variation in CC16 concentration in BALF can cause large variations in serum CC16 concentrations. Previous studies showed that NAC enhanced the production of CC16 in a mouse allergy model, a possible mechanism by which it may suppress airway inflammation [49]. In our model NAC had minor effects on lung CC16 concentrations of sensitized and challenged mice maybe because our model reproduces a mild allergic inflammation of the lung, as shown by the data on inflammatory cells, cytokines and airway hyperresponsiveness (See additional file 1: functional characterization of the mouse model). On the contrary, we show strong effects of NAC upon EC-UFP exposure.

Transmission and scanning electron microscopy served to investigate UFP-induced morphological alterations in Clara cells. EC-UFP are found in the ultrafine size range (<100 nm), which when inhaled, readily penetrate the lower respiratory tract [50]. However, we did not detect particles within Clara cells as we recently observed in other types of epithelial cells [29]. Thus, the morphological and functional alterations of Clara cells which we observed is most likely a consequence of particle indirect effects, such as alterations of inflammatory mediators in the lung lining fluid. Ultrastructural alterations of Clara cells were also shown following exposures to air pollutants such as ozone and tobacco smoke [21,51]. Acute injury to Clara cells induced by ozone exposure caused hyperplasia and loss of secretory granules [52]. Studies on CC16 -/- mice showed that CC16 is required for the appearance of Clara cells secretory granules [53]. These studies suggest an important role for Clara cells and oxidant-induced secretion of CC16 as an immediate protective response to oxidative injury. Surprisingly, we showed hyperplasia of Clara cells (but no goblet cell metaplasia) following inhalation of relatively inert particles, such as EC-UFP. Sensitized and challenged mice exposed to filtered air were characterized by increased numbers of mucous cells derived by Clara cell metaplasia, as previously described [28]. In the morphometrical analysis of PAS positive material we showed that this phenomenon was significant only in the large airways. Interestingly, sensitized and challenged mice exposed to EC-UFP prior to challenge showed increased goblet cell metaplasia induced by EC-UFP exposure only in the small airways, in contrast to large airways. One explanation could be based on predominant ultrafine particle deposition in smaller compared to larger airways due to the velocity profiles generated in the airways by high frequency breathing in rodents and the increased residence time of particles in small structures favouring deposition by diffusional displacement [54,55]. Electron miscroscopy shows most dramatic alterations of Clara cell ultrastructure in this group, characterized by extreme hyperplasia, metaplasia with complete loss of secretory granules and necrosis. We show that oxidative stress plays an important role, since NAC treatment is able to limit goblet cell metaplasia and protect Clara cell ultrastructure.

## Conclusion

Our study demonstrates the role of oxidative stress on Clara cell function and morphology in a mouse model of allergic inflammation of the lung. In healthy animals, Clara cells showed to contribute to the local needs upon cell damage caused by EC-UFP-induced oxidative stress. In allergic animals, the combination of goblet cell metaplasia, with associated mucus hypersecretion, and reduced host defence seems a disastrous pathophysiological combination, where the invoked compensatory mechanisms seem to be insufficient. For asthmatics, particle exposure would then exacerbate the already existing difficulty to counteract reactive oxygen species. Our data support the concept that allergic individuals are more susceptible to the adverse health effects of EC-UFP.

## List of Abbreviations

CC16: Clara cell protein; EC-UFP: elemental carbon ultrafine particles; UFP: ultrafine particles; OVA: ovalbumin; NAC: N-acetylcysteine; BALF: bronchoalveolar lavage fluid; TNF-α: tumor necrosis factor alpha; DEP: diesel exhaust particles; PBS: phosphate buffered saline; ELISA: enzyme-linked immunosorbent assay; BAL: bronchoalveolar lavage; RT-PCR: reverse transcription-polymerase chain reaction; PAS: periodic acid Schiff; TEM: transmission electron microscopy; SEM: scanning electron microscopy.

## Competing interests

The authors declare that they have no competing interests.

## Authors' contributions

FA and HB conceived the overall research idea, made substantial contributions to design, acquisition, analysis and interpretation of data. FA carried out all procedures for animal experiments and wrote the manuscript. IW contributed substantially to the study by performing all electron microscopy. EvM and AB performed and analysed CC16 assessment. ST performed the morphometrical analysis of the histological samples. HS performed lung function tests. EK supervised particles exposures and CB and MM supervised the gene expression analysis by real-time PCR. All authors read and approved the final manuscript.

## Supplementary Material

Additional file 1 Functional characterization of the mouse model. Click here for file

## References

[B1] Pope CA Epidemiology of fine particulate air pollution and human health: biologic mechanisms and who's at risk? Environ Health Perspect 2000 108 Suppl 4 713 23 10.2307/3454408 10931790 PMC1637679

[B2] Peters A Wichmann HE Tuch T Heinrich J Heyder J Respiratory effects are associated with the number of ultrafine particles Am J Respir Crit Care Med 1997 155 4 1376 83 9105082 10.1164/ajrccm.155.4.9105082

[B3] Li N Sioutas C Cho A Schmitz D Misra C Sempf J Wang M Oberley T Froines J Nel A Ultrafine particulate pollutants induce oxidative stress and mitochondrial damage Environ Health Perspect 2003 111 4 455 60 12676598 10.1289/ehp.6000 PMC1241427

[B4] Smith MN Greenberg SD Spjut HJ The Clara cell: a comparative ultrastructural study in mammals Am J Anat 1979 155 1 15 30 10.1002/aja.1001550103 463790

[B5] Pack RJ Al-Ugaily LH Morris G The cells of the tracheobronchial epithelium of the mouse: a quantitative light and electron microscope study J Anat 1981 132 Pt 1 71 84 7275793 PMC1233396

[B6] Massaro GD Singh G Mason R Plopper CG Malkinson AM Gail DB Biology of the Clara cell Am J Physiol 1994 266 1 Pt 1 L101 6 7905712 10.1152/ajplung.1994.266.1.L101

[B7] Bedetti CD Singh J Singh G Katyal SL Wong-Chong ML Ultrastructural localization of rat Clara cell 10 KD secretory protein by the immunogold technique using polyclonal and monoclonal antibodies J Histochem Cytochem 1987 35 7 789 94 2438324 10.1177/35.7.2438324

[B8] Singh G Katyal SL Clara cells and Clara cell 10 kD protein (CC10) Am J Respir Cell Mol Biol 1997 17 2 141 3 9271300 10.1165/ajrcmb.17.2.f138

[B9] Dierynck I Bernard A Roels H De Ley M The human Clara cell protein: biochemical and biological characterisation of a natural immunosuppressor Mult Scler 1996 1 6 385 7 9345423 10.1177/135245859600100621

[B10] Hayward A Who's to blame for asthma? Lancet 1995 346 8985 1243 10.1016/S0140-6736(95)91855-8 7475710

[B11] Levin SW Butler JD Schumacher UK Wightman PD Mukherjee AB Uteroglobin inhibits phospholipase A2 activity Life Sci 1986 38 20 1813 9 10.1016/0024-3205(86)90135-9 3084897

[B12] Yao XL Levine SJ Cowan MJ Logun C Shelhamer JH Tumor necrosis factor-alpha stimulates human Clara cell secretory protein production by human airway epithelial cells Am J Respir Cell Mol Biol 1998 19 4 629 35 9761760 10.1165/ajrcmb.19.4.3129

[B13] Lesur O Langevin S Berthiaume Y Legare M Skrobik Y Bellemare JF Levy B Fortier Y Lauzier F Bravo G Nickmilder M Rousseau E Bernard A Outcome value of Clara cell protein in serum of patients with acute respiratory distress syndrome Intensive Care Med 2006 32 8 1167 74 10.1007/s00134-006-0235-1 16794838

[B14] Bernard A Carbonnelle S Nickmilder M de Burbure C Non-invasive biomarkers of pulmonary damage and inflammation: Application to children exposed to ozone and trichloramine Toxicol Appl Pharmacol 2005 206 2 185 90 10.1016/j.taap.2004.10.022 15967207

[B15] Michel O Murdoch R Bernard A Inhaled LPS induces blood release of Clara cell specific protein (CC16) in human beings J Allergy Clin Immunol 2005 115 6 1143 7 10.1016/j.jaci.2005.01.067 15940126

[B16] Bernard AM Roels HA Buchet JP Lauwerys RR Serum Clara cell protein: an indicator of bronchial cell dysfunction caused by tobacco smoking Environ Res 1994 66 1 96 104 10.1006/enrs.1994.1047 8013441

[B17] Van Miert E Dumont X Bernard A CC16 as a marker of lung epithelial hyperpermeability in an acute model of rats exposed to mainstream cigarette smoke Toxicol Lett 2005 159 2 115 23 10.1016/j.toxlet.2005.05.007 16165332

[B18] Arsalane K Broeckaert F Knoops B Clippe A Buchet JP Bernard A Increased serum and urinary concentrations of lung clara cell protein in rats acutely exposed to ozone Toxicol Appl Pharmacol 1999 159 3 169 74 10.1006/taap.1999.8738 10486303

[B19] Gowdy K Krantz QT Daniels M Linak WP Jaspers I Gilmour MI Modulation of pulmonary inflammatory responses and antimicrobial defenses in mice exposed to diesel exhaust Toxicol Appl Pharmacol 2008 229 3 310 9 10.1016/j.taap.2008.01.040 18343473

[B20] Mango GW Johnston CJ Reynolds SD Finkelstein JN Plopper CG Stripp BR Clara cell secretory protein deficiency increases oxidant stress response in conducting airways Am J Physiol 1998 275 2 Pt 1 L348 56 9700096 10.1152/ajplung.1998.275.2.L348

[B21] Plopper CG Mango GW Hatch GE Wong VJ Toskala E Reynolds SD Tarkington BK Stripp BR Elevation of susceptibility to ozone-induced acute tracheobronchial injury in transgenic mice deficient in Clara cell secretory protein Toxicol Appl Pharmacol 2006 213 1 74 85 10.1016/j.taap.2005.09.003 16226776

[B22] Bochner BS Busse WW Allergy and asthma J Allergy Clin Immunol 2005 115 5 953 9 10.1016/j.jaci.2005.02.032 15867851

[B23] Van Vyve T Chanez P Bernard A Bousquet J Godard P Lauwerijs R Sibille Y Protein content in bronchoalveolar lavage fluid of patients with asthma and control subjects J Allergy Clin Immunol 1995 95 1 Pt 1 60 8 10.1016/S0091-6749(95)70153-2 7822665

[B24] Laing IA Hermans C Bernard A Burton PR Goldblatt J Le Souef PN Association between plasma CC16 levels, the A38G polymorphism, and asthma Am J Respir Crit Care Med 2000 161 1 124 7 10619808 10.1164/ajrccm.161.1.9904073

[B25] Shijubo N Itoh Y Yamaguchi T Sugaya F Hirasawa M Yamada T Kawai T Abe S Serum levels of Clara cell 10-kDa protein are decreased in patients with asthma Lung 1999 177 1 45 52 10.1007/PL00007626 9835633

[B26] Hung CH Chen LC Zhang Z Chowdhury B Lee WL Plunkett B Chen CH Myers AC Huang SK Regulation of TH2 responses by the pulmonary Clara cell secretory 10-kd protein J Allergy Clin Immunol 2004 114 3 664 70 10.1016/j.jaci.2004.05.042 15356574

[B27] Kuperman DA Huang X Nguyenvu L Holscher C Brombacher F Erle DJ IL-4 receptor signaling in Clara cells is required for allergen-induced mucus production J Immunol 2005 175 6 3746 52 16148120 10.4049/jimmunol.175.6.3746

[B28] Reader JR Tepper JS Schelegle ES Aldrich MC Putney LF Pfeiffer JW Hyde DM Pathogenesis of mucous cell metaplasia in a murine asthma model Am J Pathol 2003 162 6 2069 78 12759261 10.1016/S0002-9440(10)64338-6 PMC2216702

[B29] Alessandrini F Beck-Speier I Krappmann D Weichenmeier I Takenaka S Karg E Kloo B Schulz H Jakob T Mempel M Behrendt H Role of Oxidative Stress in Ultrafine Particle-Induced Exacerbation of Allergic Lung Inflammation Am J Respir Crit Care Med 2009 179 11 984 91 10.1164/rccm.200807-1061OC 19264975

[B30] Alessandrini F Schulz H Takenaka S Lentner B Karg E Behrendt H Jakob T Effects of ultrafine carbon particle inhalation on allergic inflammation of the lung J Allergy Clin Immunol 2006 117 4 824 30 10.1016/j.jaci.2005.11.046 16630940

[B31] Su DS Muller JO Jentoft RE Rothe D Jacob E Schlogl R Fullerene-like soot from EuroIV diesel engine: consequences for catalytic automotive pollution control Topics in Catalysis 2004 30-31 1-4 241 245 10.1023/B:TOCA.0000029756.50941.02

[B32] Matuschek G Karg E Schroppel A Schulz H Schmid O Chemical investigation of eight different types of carbonaceous particles using thermoanalytical techniques Environ Sci Technol 2007 41 24 8406 11 10.1021/es062660v 18200871

[B33] Halatek T Hermans C Broeckaert F Wattiez R Wiedig M Toubeau G Falmagne P Bernard A Quantification of Clara cell protein in rat and mouse biological fluids using a sensitive immunoassay Eur Respir J 1998 11 3 726 33 9596129

[B34] Weibel E Stereological Methods: Practical methods for biological morphometry 1979 1 London: Academic

[B35] Schwartz J Slater D Larson TV Pierson WE Koenig JQ Particulate air pollution and hospital emergency room visits for asthma in Seattle Am Rev Respir Dis 1993 147 4 826 31 8466116 10.1164/ajrccm/147.4.826

[B36] de Haar C Hassing I Bol M Bleumink R Pieters R Ultrafine carbon black particles cause early airway inflammation and have adjuvant activity in a mouse allergic airway disease model Toxicol Sci 2005 87 2 409 18 10.1093/toxsci/kfi255 16014737

[B37] von Klot S Wolke G Tuch T Heinrich J Dockery DW Schwartz J Kreyling WG Wichmann HE Peters A Increased asthma medication use in association with ambient fine and ultrafine particles Eur Respir J 2002 20 3 691 702 10.1183/09031936.02.01402001 12358349

[B38] Gangl K Reininger R Bernhard D Campana R Pree I Reisinger J Kneidinger M Kundi M Dolznig H Thurnher D Valent P Chen KW Vrtala S Spitzauer S Valenta R Niederberger V Cigarette smoke facilitates allergen penetration across respiratory epithelium Allergy 2009 64 3 398 405 10.1111/j.1398-9995.2008.01861.x 19120070

[B39] Morimoto Y Ding L Oyabu T Hirohashi M Kim H Ogami A Yamato H Akiyama I Hori H Higashi T Tanaka I Expression of Clara cell secretory protein in the lungs of rats exposed to silicon carbide whisker in vivo Toxicol Lett 2003 145 3 273 9 10.1016/S0378-4274(03)00308-4 14580898

[B40] Cao Q Zhang S Dong C Song W Pulmonary responses to fine particles: differences between the spontaneously hypertensive rats and wistar kyoto rats Toxicol Lett 2007 171 3 126 37 10.1016/j.toxlet.2007.05.007 17606336

[B41] Hay JG Danel C Chu CS Crystal RG Human CC10 gene expression in airway epithelium and subchromosomal locus suggest linkage to airway disease Am J Physiol 1995 268 4 Pt 1 L565 75 7733299 10.1152/ajplung.1995.268.4.L565

[B42] Vilcek J Lee TH Tumor necrosis factor. New insights into the molecular mechanisms of its multiple actions J Biol Chem 1991 266 12 7313 6 1850405

[B43] Finkelstein JN Johnston C Barrett T Oberdorster G Particulate-cell interactions and pulmonary cytokine expression Environ Health Perspect 1997 105 Suppl 5 1179 82 10.2307/3433529 9400720 PMC1470156

[B44] Yamamoto S Tin Tin Win S Ahmed S Kobayashi T Fujimaki H Effect of ultrafine carbon black particles on lipoteichoic acid-induced early pulmonary inflammation in BALB/c mice Toxicol Appl Pharmacol 2006 213 3 256 66 10.1016/j.taap.2005.11.007 16387335

[B45] Lensmar C Nord M Gudmundsson GH Roquet A Andersson O Jornvall H Eklund A Grunewald J Agerberth B Decreased pulmonary levels of the anti-inflammatory Clara cell 16 kDa protein after induction of airway inflammation in asthmatics Cell Mol Life Sci 2000 57 6 976 81 10.1007/PL00000738 10950311 PMC11147083

[B46] Aruoma OI Halliwell B Hoey BM Butler J The antioxidant action of N-acetylcysteine: its reaction with hydrogen peroxide, hydroxyl radical, superoxide, and hypochlorous acid Free Radic Biol Med 1989 6 6 593 7 10.1016/0891-5849(89)90066-X 2546864

[B47] Rahman I MacNee W Oxidative stress and regulation of glutathione in lung inflammation Eur Respir J 2000 16 3 534 54 10.1034/j.1399-3003.2000.016003534.x 11028671

[B48] Moldeus P Cotgreave IA N-acetylcysteine Methods Enzymol 1994 234 482 92 full_text 7808322 10.1016/0076-6879(94)34119-2

[B49] Nie X Li Q Cai G Dai Y Zhang J The effect of N-acetylcysteine on Clara cells and Clara cell 16 kDa protein in a murine model of allergen-induced airway inflammation Respirology 2005 10 2 157 63 10.1111/j.1440-1843.2005.00698.x 15823179

[B50] Oberdorster G Utell MJ Ultrafine particles in the urban air: to the respiratory tract--and beyond? Environ Health Perspect 2002 110 8 A440 1 12153769 10.1289/ehp.110-1240959 PMC1240959

[B51] Van Winkle LS Evans MJ Brown CD Willits NH Pinkerton KE Plopper CG Prior exposure to aged and diluted sidestream cigarette smoke impairs bronchiolar injury and repair Toxicol Sci 2001 60 1 152 64 10.1093/toxsci/60.1.152 11222882

[B52] Evans MJ Oxidant gases Environ Health Perspect 1984 55 85 95 10.2307/3429694 6376113 PMC1568353

[B53] Stripp BR Reynolds SD Boe IM Lund J Power JH Coppens JT Wong V Reynolds PR Plopper CG Clara cell secretory protein deficiency alters clara cell secretory apparatus and the protein composition of airway lining fluid Am J Respir Cell Mol Biol 2002 27 2 170 8 12151308 10.1165/ajrcmb.27.2.200200270c

[B54] Slutsky AS Kamm RD Drazen JM Engel LA, Paiva M Alveolar ventilation at high frequencies using tidal volumes smaller than the anatomical dead space Gas mixing and distribution in the lung 1985 Marcel Dekker, Inc, New York, Basel 137 176

[B55] Schulz H Brand P Heyder J Gehr P, Heyder J Particle deposition in the respiratory tract Particle-lung interactions 2000 Marcel Dekker, Inc, New York, Basel 229 290

