# Supplementary material for: Effects of ultrafine particles-induced oxidative stress on Clara cells in allergic lung inflammation
Source: Part Fibre Toxicol. 2010 Apr 26;7:11. doi: 10.1186/1743-8977-7-11 (PMC2880284; doi:10.1186/1743-8977-7-11)
Supplement: Additional file 1 — Functional characterization of the mouse model. [file 1743-8977-7-11-S1.PDF]

Effects of Ultrafine Particles-induced Oxidative Stress on Clara Cells in Allergic Lung Inflammation

Francesca Alessandrini, Ingrid Weichenmeier, Erik van Miert, Shinji Takenaka, Erwin Karg, Cornelia Blume, Martin Mempel, Holger Schulz, Alfred Bernard, Heidrun Behrendt

Additional file 1: functional characterization of the mouse model

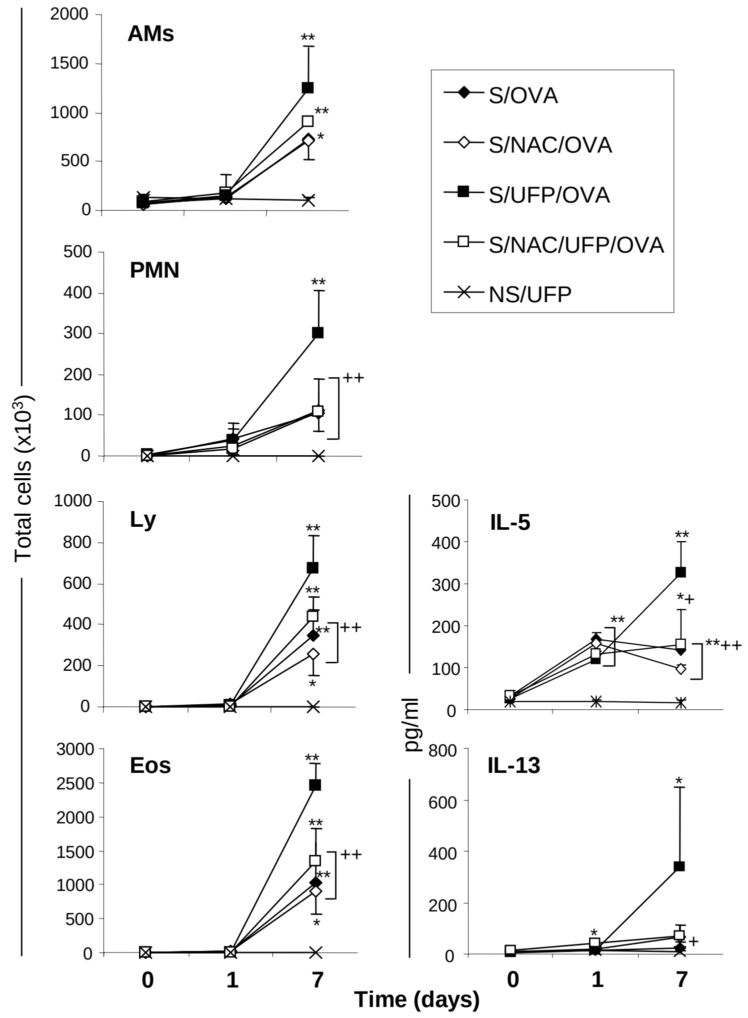

Fig. 1: BAL Cells and Cytokines. BAL cells (**left**) and BAL fluid cytokines (**right**) in sensitized and challenged mice (S/OVA), treated with NAC (S/NAC/OVA), or exposed to EC-UFP 24h prior to OVA challenge (S/UFP/OVA), and treated with NAC prior to and close

to mid EC-UFP exposure (S/NAC/UFP/OVA). Non sensitized mice exposed to EC-UFP (NS/UFP) served as controls. Data presented as mean ± SD (n=4/time point). AMs, alveolar macrophages; PMN, polymorphonuclear neutrophils; Ly, lymphocytes; Eos, eosinophils. *p<0.05, **p<0.01 vs NS/UFP; +p<0.05, ++p<0.01 vs S/UFP/OVA.

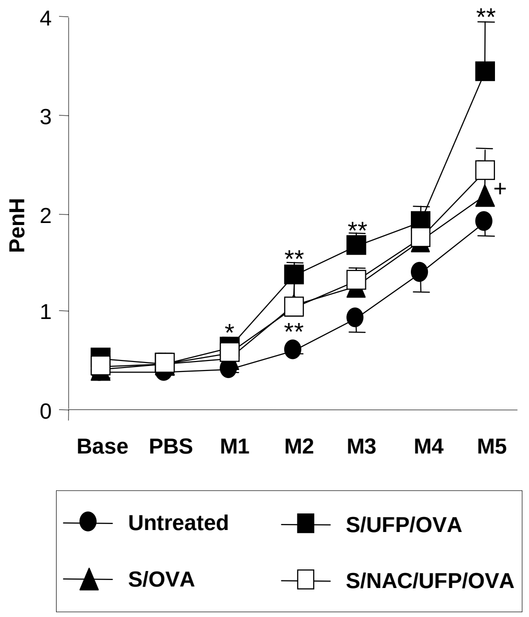

Fig. 2: Airway hyperresponsiveness, represented by PenH, measured with a non-invasive body plethysmograph 24 h after OVA challenge. Untreated mice (untreated), OVA sensitized and challenged mice (S/OVA), OVA sensitized mice exposed to EC-UFP 24 h prior to OVA challenge (S/UFP/OVA) and OVA sensitized mice exposed to EC-UFP 24 h prior to OVA challenge treated with NAC (S/NAC/UFP/OVA). Data are expressed as mean ± SEM; n=5-7/ group. *p<0.05, **p<0.01 vs untreated; +p=0.05 vs S/UFP/OVA.
